# Notes on Health Resorts

**Published:** 1899-08-12

**Authors:** 


### The Institutional Workshop.

### NOTES ON HEALTH RESORTS.

### HARROGATE.

Harrogate is a town which appeals to the public in several capacities. On the one hand it is a residential town, practically a well-to-do suburb of Leeds, from which it can be reached in about half-an-hour. Again, as the result of its clear and bracing air, its parks and gardens, the beautiful drives which can be had on every side, and the good hotels with which it is provided, it has become a great pleasure resort. Then for much the same reasons, and because it stands high and dry and so invites people to be much in the open air, it has become a tonic health resort; and, lastly, by virtue of its sulphur springs, it has long been famous all the world over as a place for the treatment of disease by means of its baths and its waters. In regard to its position as a general tonic health resort we need not say much. Its climate is, as the guide books say, "dry and bracing;" it stands at a fair altitude, from 300 to 450 feet above the sea level; its mortality is low, epidemic diseases are said to be rare, its water supply is soft, pure, and abundant, and every facility and attraction is provided for outdoor exercises and amusements, and, we may add, that in winter it is largely resorted to as a fox-hunting centre.

As a health resort for the treatment of debility, anæmia, and the fag of town life, for cases with a tendency to tuberculosis, and for the management of convalescence, Harrogate is then not only an attractive but a most useful place.

It is, however, in connection with its waters that Harrogate is best known, and although we do not feel inclined to attribute to them, so exclusively as some people do, all the benefits which the patients using them evidently derive from visiting Harrogate, everyone must admit their utility and their power for good in proper cases.

If we are to judge of mineral waters and their actions impartially, we must regard them from a somewhat broader point of view than is apt to be taken by those who are interested in any particular spa, and when we see patients rapidly recovering under the use of waters which, from their strong mineralisations, may be looked upon as somewhat active drugs, we must not forget the scores of other cases, not unlike in nature, which are also to be seen recovering at other spas whose waters smack of *aqua pura* rather than of the drug shop.

At such spas, where the water is found upon analysis to be merely impregnated with a small quantity of some earthy salts, we are bound to fall back upon the surroundings, and to say that the change of habits, the open air life, the regulated exercise, the early hours, the bathing and shampooing, as well as the washing out of the system by the free draughts of water, are in themselves sufficient to account for the cures which undoubtedly abound; and when we come to Harrogate and find patients doing the same things and using all the same aids to recovery which people enjoy at other spas, and getting well, we must not, because they also drink large doses of medicinal water twice a day, jump to the conclusion, as so many do, that it is the Harrogate water which does the good. Plenty of people, even people with bad livers, get well at Harrogate, who would probably have done just as well if, along with the general and bath treatment, they had merely drunk freely of the pure water laid on by the corporation. But there is a fair residuum to whom the waters of Harrogate are definitely advantageous, and who derive a benefit from them outside and beyond all that they can find at other spas, and this fact makes it desirable to consider shortly of what the Harrogate waters are composed, and in what diseases they are found most effectual. Broadly speaking, all the saline matters usually met with in medicinal springs, except arsenic, are to be found in one or other of the Harrogate waters, generally mingled (and always in the case of the sulphur springs) with considerable quantities of chlorides, especially common salt. The waters may be divided into two main groups, the sulphur group and the iron group.

#### (1) The Sulphur Group.

The sulphur is met with in two forms, in some wells as a sodium sulphydrate, with free sulphuretted hydrogen; in others as a sodium sulphide. The typical sulphur waters are, according to their dose and the times at which they are taken, laxative, aperient, or purgative; they are also diuretic. They are largely used in various forms of indigestion, especially when this is accompanied with constipation, flatulence, and acidity; and although it can hardly be said that in the treatment of such cases they are essential, still there can be no doubt that much time is often saved by their use, so that patients get over the first slow step towards recovery much more quickly than they would do by merely dietetic and hygienic treatment. It is in liver cases, however, that the sulphur waters seem to show their powers with greatest effect. Town dwellers, people who with active minds find their bodies getting stout and torpid, business men who have driven themselves on through business hours with repeated nips of alcohol, and people with a moderate amount of intermittent catarrh of the bile passages, and its accompanying jaundice; all these people find immense relief from the use of one or other of the sulphur waters. Then all forms of chronic catarrh seem to be more or less influenced by these waters; certainly stomachic and bronchial catarrhs are often much relieved by their use even in the winter. Chronic skin diseases, again, frequently derive much benefit, sometimes from the baths, sometimes from drinking the waters. In fact, Harrogate becomes at certain times of the year a perfect museum of skin diseases and yellow faces.

#### (2) The Iron Group.

One of the great advantages which Harrogate possesses is that, without materially altering some of the most active agents in the waters which are being employed, it is possible, by turning from the sulphur to the saline chalybeate waters, to introduce a tonic in place of an alterative influence. But in addition to serving as an after-cure in cases which have already gone through an eliminative or alterative course, the chalybeate waters have their own special efficacy in the treatment of a great variety of cases of anæmia. A large number of cases of chlorosis and of general debility derive much benefit from visiting Harrogate, and this not entirely from the waters, useful as they are in such cases. The surroundings of the place, the many temptations to take exercise, the riding and the cycling, all help to drive away that torpidity of system which so greatly favours the long continuance of chlorosis. The waters, however, are of great importance in such cases, the more aperient waters cutting at the root of the copræmia which is often at the bottom of the disease; while the iron waters are of peculiar efficacy in restoring the condition of the blood. The iron waters all contain the ferrous carbonate, and the chloride of iron spring contains a goodly dose of ferrous chloride in addition. How far their action is helped by the added sodium chloride is perhaps difficult to say; but it does seem to be the fact that these salty iron waters are more easily digested than some which appear to be of a more simple constitution. It is just possible that the barium which some of these waters contain may have a good effect on the dilated and feeble hearts which so many chlorotics possess, while the calcium may also be of some assistance in other cases, and, of course, the possibility of alternating the iron with the more definitely aperient and alterative waters gives to the bath physician practising at Harrogate a large choice of therapeutic measure with which to meet the various complications of chlorosis. In regard to the barium which is met with in still larger proportion in some of the sulphur group of waters, it is held by some that it has a specially tonic action on the heart, and that to its presence is due the fact, on which considerable stress is laid, that even a free purgation by Harrogate sulphur water does not have that depressing effect which the same amount of evacuation by other means is apt to produce.

Cases of gout, of rheumatism, and of rheumatic gout, notwithstanding the differences which exist between them, agree in this that they are in many cases much relieved by treatment at Harrogate, and perhaps the reason is not far to seek. The waters are in essence alterative, purgative, and diuretic; and, different as may be the poisons engaged, there can be but little doubt that many cases of each of these diseases have their origin in one or another form of toxæmia; so that we need not be surprised at hearing that they derive a considerable amount of benefit from the eliminative waters of this place. It is more especially in regard to such cases, and to those of heart disease which now come for the Nauheim treatment, that the large expenditure which the Corporation of Harrogate has lately incurred in the enlargement and improvement of the baths is likely to be remunerative. The arrangements now available for the treatment of rheumatic cases, by baths, massage, massage douches, and, lastly, by hot dry air, are very complete.

The after effects of lead poisoning, a moderate degree of which is by no means uncommon among those who drink the soft water supplied to many northern towns, derive much benefit from treatment at Harrogate, as also do those of syphilis. Altogether, taking Harrogate as what the Germans call a "cure," it is one of the most important, if not the most important of which England can boast. Nevertheless, the very variety of the resources which it offers for the treatment of disease entails this—drawback some people would call it, but advantage as it seems to us—that no one can attempt to treat himself, so great is the multitude of waters. It is like being turned loose in a chemist's shop and asked to take one's choice. So one has perforce to consult a physician. In places where there is only one water people sometimes think there is nothing to do but take it. So they take it to their own undoing. In Harrogate things are different; medical guidance is essential, and perhaps to this is due some of its success.

